# EndMT Regulation by Small RNAs in Diabetes-Associated Fibrotic Conditions: Potential Link With Oxidative Stress

**DOI:** 10.3389/fcell.2021.683594

**Published:** 2021-05-19

**Authors:** Roberta Giordo, Yusra M. A. Ahmed, Hilda Allam, Salah Abusnana, Lucia Pappalardo, Gheyath K. Nasrallah, Arduino Aleksander Mangoni, Gianfranco Pintus

**Affiliations:** ^1^Department of Medical Laboratory Sciences, College of Health Sciences and Sharjah Institute for Medical Research, University of Sharjah, Sharjah, United Arab Emirates; ^2^Department of Diabetes and Endocrinology, University Hospital Sharjah, Sharjah, United Arab Emirates; ^3^Department of Clinical Sciences, College of Medicine, University of Sharjah, Sharjah, United Arab Emirates; ^4^Department of Biology, Chemistry and Environmental Studies, American University of Sharjah, Sharjah, United Arab Emirates; ^5^Department of Biomedical Sciences, College of Health Sciences Member of QU Health, Qatar University, Doha, Qatar; ^6^Biomedical Research Center, Qatar University, Doha, Qatar; ^7^Discipline of Clinical Pharmacology, College of Medicine and Public Health, Flinders University, Adelaide, SA, Australia; ^8^Flinders Medical Centre, Adelaide, SA, Australia; ^9^Department of Biomedical Sciences, University of Sassari, Sassari, Italy

**Keywords:** EndMT, miRNAs, diabetes, fibrosis, oxidative stress

## Abstract

Diabetes-associated complications, such as retinopathy, nephropathy, cardiomyopathy, and atherosclerosis, the main consequences of long-term hyperglycemia, often lead to organ dysfunction, disability, and increased mortality. A common denominator of these complications is the myofibroblast-driven excessive deposition of extracellular matrix proteins. Although fibroblast appears to be the primary source of myofibroblasts, other cells, including endothelial cells, can generate myofibroblasts through a process known as endothelial to mesenchymal transition (EndMT). During EndMT, endothelial cells lose their typical phenotype to acquire mesenchymal features, characterized by the development of invasive and migratory abilities as well as the expression of typical mesenchymal products such as α-smooth muscle actin and type I collagen. EndMT is involved in many chronic and fibrotic diseases and appears to be regulated by complex molecular mechanisms and different signaling pathways. Recent evidence suggests that small RNAs, in particular microRNAs (miRNAs) and long non-coding RNAs (lncRNAs), are crucial mediators of EndMT. Furthermore, EndMT and miRNAs are both affected by oxidative stress, another key player in the pathophysiology of diabetic fibrotic complications. In this review, we provide an overview of the primary redox signals underpinning the diabetic-associated fibrotic process. Then, we discuss the current knowledge on the role of small RNAs in the regulation of EndMT in diabetic retinopathy, nephropathy, cardiomyopathy, and atherosclerosis and highlight potential links between oxidative stress and the dyad small RNAs-EndMT in driving these pathological states.

## Introduction

Diabetes mellitus (DM) is one of the most common chronic diseases worldwide (Lin X. et al., 2020). A prediction study estimated a significant further increase in the number of people suffering from diabetes, especially in developing countries, with a global prevalence of 7.7% (439 million adults) by 2030 (Shaw et al., 2010; Lin X. et al., 2020). Long-term hyperglycemia is the main driver of the onset and the progression of common diabetic complications, particularly those affecting the eye, kidney, nervous system, and cardiovascular system (Deshpande et al., 2008). Such complications are secondary to structural and functional alterations of organs and tissues that are caused by an increased cellular glucose uptake (Wellen and Hotamisligil, 2005). This activates inflammatory pathways which ultimately leads to excessive deposition of extra cellular matrix (ECM) proteins and consequent thickening of the vessel wall (Wellen and Hotamisligil, 2005; Wynn, 2008). Tissue fibrosis is therefore the common denominator of most diabetic complications, including atherosclerosis, cardiomyopathy, nephropathy and retinopathy (Ban and Twigg, 2008). Myofibroblasts are the key mediators of pathological ECM accumulation (Kendall and Feghali-Bostwick, 2014). These cells are normally involved in tissue repair and are subsequently removed by apoptosis at the end of the repair process. However, under pathological situations, their unrestrained activation leads to excessive ECM deposition (Micallef et al., 2012). Myofibroblasts originate from different precursor cells, depending on the organ and the type of initial injury (Bochaton-Piallat et al., 2016). Although fibroblasts represent the primary source of myofibroblasts, the latter can also originate from the inresident or bone marrow-derived mesenchymal cells as well as epithelial and endothelial cells (ECs), through a process known as epithelial/endothelial to mesenchymal transition (Micallef et al., 2012; Kendall and Feghali-Bostwick, 2014). In particular, endothelial to mesenchymal transition (EndMT), the process involving ECs, is emerging as an important player in the pathogenesis of diabetic fibrosis (Srivastava et al., 2013; Cao et al., 2014; Souilhol et al., 2018). ECs, constituting the inner layer of blood vessels, are responsible for maintaining vascular homeostasis in response to endogenous and exogenous perturbations (Sandoo et al., 2010; Khaddaj Mallat et al., 2017). There is good evidence that ECs, when exposed to hyperglycemia, undergo significant alterations that result in an imbalance between vasodilation and vasoconstriction as well as the development of inflammatory and vascular complications (Bakker et al., 2009; Meza et al., 2019). Moreover, high glucose concentrations have been shown to trigger the shift of the endothelium toward the mesenchymal phenotype (Yu et al., 2017; Giordo et al., 2021). Overall, EndMT appears to represent the key link in the interaction between inflammation and endothelial dysfunction in diabetic complications (Cho et al., 2018; Man et al., 2019). In the setting of EndMT, ECs lose their typical cobblestone morphology and tight junctions and acquire increased motility and the ability to secrete ECM proteins (Dejana et al., 2017). In addition, concurrently with the loss of typical endothelial markers, such as vascular endothelial cadherin (VE-cadherin), platelet endothelial cell adhesion molecule (PECAM-1), also known as CD31, and von Willebrand Factor (vWF), they acquire the ability to express several mesenchymal markers, such as alpha-smooth muscle actin (α-SMA), smooth muscle protein 22 alpha (SM22α), fibronectin, vimentin, and fibroblast specific protein-1 (FSP-1) (Dejana et al., 2017; Hong et al., 2018). EndMT is involved in many chronic and fibrotic disease states and appears to be regulated by several factors (Evrard et al., 2016; Thuan et al., 2018; Phan et al., 2020). In diabetes, oxidative stress is emerging as an important trigger of the ECs transformation into myofibroblasts and vascular remodeling (Montorfano et al., 2014; Thuan et al., 2018). Indeed, hyperglycemia can increase the production of reactive oxygen species (ROS), which in turn activate signaling pathways leading to the disruption of ECs hemostasis (Russell et al., 2002; Peng et al., 2013; Li et al., 2017; Volpe et al., 2018). Several signaling pathways have been demonstrated to be involved in EndMT regulation, e.g., transforming growth factor-beta (TGF-β) signaling, Notch signaling, fibroblast growth factor/fibroblast growth factor receptor 1 (FGF/FGFR1) signaling pathway, Smad2/3-mediated pathways (Piera-Velazquez and Jimenez, 2019) and pro-inflammatory signaling cascades (Lin et al., 2018; Ferreira et al., 2019). An important role in the regulation of EndMT is also played by micro RNAs (miRNAs), a class of short endogenous non-coding RNAs that regulate gene expression at post-transcriptional level by binding to the 3′-untranslated region of messenger RNA (mRNA) (Kim et al., 2015; Michlewski and Cáceres, 2019). A single miRNA can target multiple mRNAs, thus influencing several processes such as cell differentiation, proliferation, and apoptosis (Vidigal and Ventura, 2015). miRNAs can also target significant parts of pathways since miRNAs with similar (seed) sequence target similar sets of genes and thus similar sets of pathways (Kehl et al., 2017). Moreover miRNAs can, either positively or negatively, regulate gene expression (Catalanotto et al., 2016). As a result, they represent promising markers and druggable targets for many diseases, including diabetes (Regazzi, 2018; Cao et al., 2019; Fan et al., 2020). An increasing amount of evidence also suggests that diabetes progression is linked to the alteration of miRNAs expression profiles; indeed, profibrotic miRNAs, such as miR-125b, let-7c, let-7g, miR-21, miR-30b, and miR-195 have been shown to be upregulated in EndMT. By contrast, antifibrotic miRNAs, such as miR-122a, miR-127, miR-196, and miR-375, with inhibitory action toward genes responsible for EndMT, have been shown to be downregulated (Ghosh et al., 2012; Kim, 2018; Srivastava et al., 2019). In addition to miRNAs, recent studies have also demonstrated the involvement of another class of small RNAs, known as long non-coding RNAs (lncRNAs), in diabetes-associated EndMT (Feng et al., 2017; Leung and Natarajan, 2018). Compared to miRNAs, the concentrations of lncRNAs are almost tenfold lower, with the latter exhibiting significant tissue and cell specificity (Cabili et al., 2011). However, the knowledge of the function and the regulation of lncRNAs are still limited. This review aims to summarize and discuss the available knowledge on the role of small RNAs in the regulation of EndMT in diabetes-associated fibrotic complications such as retinopathy, nephropathy, cardiomyopathy, atherosclerosis, and its potential link with oxidative.

## Diabetic Nephropathy

Diabetic nephropathy (DN) is the leading cause of chronic kidney disease in about 40% of patients with type 1 and type 2 diabetes (Gross et al., 2005). Poorly controlled blood glucose concentrations can damage the filtering functionality of the kidneys, which become unable to remove waste products and extra fluids from the body (Rheinberger and Böger, 2014; Ruiz-Ortega et al., 2020). The symptoms of DN do not generally manifest in the early stages, but rather when kidney function has significantly deteriorated (Lim, 2014). Therefore, a tight blood glucose control is key to prevent the onset and progression of DN (Lewis and Maxwell, 2014; Ruiz-Ortega et al., 2020). The progression of DN is defined by various clinical stages which reflect the gradual involvement of tissue damage to different kidney compartments: glomerulus, tubules, vasculature and interstitium (Mogensen et al., 1983). The final stage of DN is characterized by renal fibrosis and organ failure, which are the result of the excessive accumulation of ECM (Calle and Hotter, 2020). Renal fibrosis is driven by multiple mechanisms, including glucose metabolism abnormalities associated with oxidative stress, inflammatory processes, and hemodynamic changes (Brosius, 2008). Consequently, many signaling pathways and cell types (mesangial cells, endothelial cells and podocytes) are involved in the fibrotic process (Badal and Danesh, 2014; Aghadavoud et al., 2017). As mentioned above, alterations of glucose metabolism not only activate various signaling pathways (Badal and Danesh, 2014; Aghadavoud et al., 2017) but also induce oxidative stress, a key pathophysiological step in the onset and progression of diabetes-associated vascular complications (Kashihara et al., 2010; Mima, 2013; Oguntibeju, 2019). Indeed, high glucose concentrations activate the diacylglycerol-protein kinase C (DAG-PKC) pathway, which is associated with endothelial dysfunction, increased production of extracellular matrix and activation of cytokines and transforming growth factor-β (TGF-β) (Koya and King, 1998; Evcimen and King, 2007). In addition, protein kinase C (PKC) induces oxidative stress by activating mitochondrial NADPH oxidase (Chen et al., 2014; Giordo et al., 2021). Increased glucose can also activate aldose reductase and the polyol pathway, leading to the depletion of Nicotinamide Adenine Dinucleotide Phosphate (NADPH), which is also required for the generation of the cellular antioxidant nitric oxide (NO) (Tesfamariam, 1994; Hummel et al., 2006; Ying, 2008; Zhao et al., 2008). The reduced NO availability compromises the balance between ROS generation and antioxidant defense, one of the leading causes of endothelial dysfunction (Schiffrin, 2008). Furthermore, hyperglycemia enhances the formation of advanced glycation end products (AGEs), proteins or lipids that become glycated as a result of exposure to sugars (Goldin et al., 2006). AGEs increase ROS production and promote inflammation and fibrosis through the activation of PKC, the nuclear factor kappa light chain enhancer of activated B cells (NF-kB) and TGF-β (Aghadavoud et al., 2017; Rhee and Kim, 2018). Within the hemodynamic factors driving renal fibrosis, an important role is played by the over-activation of the renin-angiotensin-aldosterone system (RAAS), a crucial hormone system in blood pressure regulation and fluid balance (Benigni et al., 2010; Patel et al., 2017). Hyperglycemia and insulin resistance increases the release of angiotensin II (Ang II) a potent vasoconstrictor belonging to the RAAS system (Giacchetti et al., 2005; Benigni et al., 2010; Williams and Scholey, 2018). Angiotensin II plays an important role in renal fibrosis by activating a number of factors responsible for ECM production such as TGF-β, PKC and NF-κB (Badal and Danesh, 2014; Aghadavoud et al., 2017). On the other hand, Angiotensin-converting enzyme2 (ACE2), the main modulator of the RAAS system (Benigni et al., 2010), prevents the accumulation of Ang II by catalyzing the conversion of Ang II into the vasodilator Angiotensin I (Ang I) (Batlle et al., 2010; Williams and Scholey, 2018). Although no cure is available for DN, the control of blood sugar levels and blood pressure, together with a healthy lifestyle, can slow or stop its progression. The most common DN treatments are based on the RAAS system inactivation; precisely with the use of either the ACE inhibitors (ACEis) or angiotensin receptor blockers (ARBs) or their combination (Anand and Tamura, 2012; Pathak and Dass, 2015). This type of treatments allows the lowering of proteinuria and the blood pressure within the glomerular capillaries. In addition, ACEis can also ameliorates kidney fibrosis in combination with other drugs. Is this the case of *N*-acetyl-seryl-aspartyl-lysyl-proline (AcSDKP) an antifibrotic peptide that, in combination with the ACEi, imidapril, improves kidney fibrosis restoring antifibrotic miRNAs, such as miR-29 and miR-let-7 and increasing the inhibition of the profibrotic dipeptidyl peptidase-4 (DPP-4) (Nitta et al., 2016; Srivastava et al., 2020a). DPP-4 inhibitors are another class of medicines used for DN’s treatment. In this context, due to the highest affinity for DPP-4, the drug Linagliptin is one of the most widely used (Kanasaki, 2018). In addition, promising data also come from treatments aiming at restoring Sirtuin 3 (SIRT3), which appear to ameliorate renal damage, via inhibition of aberrant glycolysis and preserving mitochondrial homeostasis (Srivastava et al., 2018; Locatelli et al., 2020).

## miRNAs Regulation of DN-Associated EndMT

**FIGURE 1:**
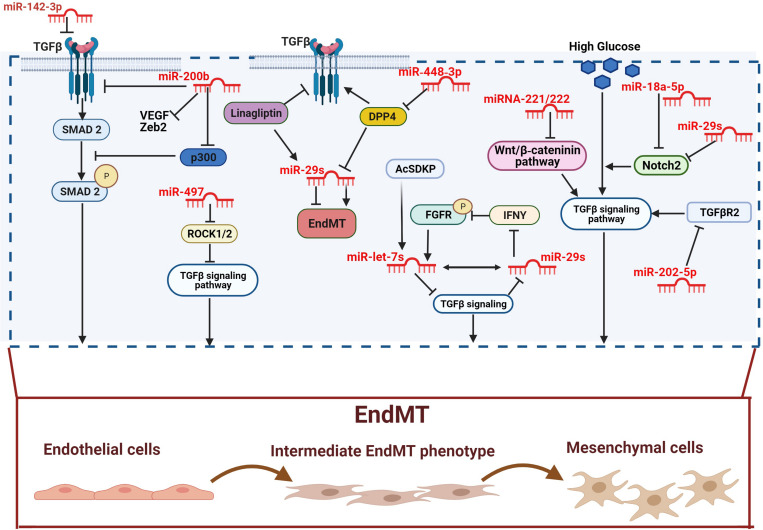
Anti-fibrotic miRNAs in diabetic complications. miR-142-3p and miR-200b inhibit EndMT by inactivating the TGF-β-SMAD pathway. The antifibrotic activity of miR-200b is played by down-regulating the TGF-β/SMAD pathway coactivator p300. miR-497 suppresses TGF-β-induced EndMT by ROCK1 and ROCK2 inactivation. The overexpression of DPP-4 is associated with the suppression of the miR-29s family anti-fibrotic activity. However, both linagliptin and AcSDKP suppresses EndMT by restoring miR-29 and miR-let-7s activities. Furthermore, miR-448-3p inhibits EndMT via DPP-4 suppression. AcSDKP upregulates the antifibrotic miR-let-7 which suppresses TGFβR1 and TGFβ signaling. The block of TGFβ signaling results in up-regulation of miR-29 gene expression, which in turn causes FGFR1 phosphorylation. FGFR1 phosphorylation is critical for miR-let-7 production. miR-29 can also target the profibrotic IFNY blocking its inhibitory action toward FGFR1. The miR-29s family inhibits high glucose-induced EndMT by down-regulating Notch2, which is also suppressed by miR-18a-5p. However, DPP-4 inhibitor and AcSDKP suppresses EndMT by restoring of miR-29 and miR-let-7s activities. Furthermore, miR-448-3p inhibit EndMT via DPP-4 suppression. The miR-29s family inhibits high glucose-induced EndMT by the downregulation of Notch2 which is also suppressed by miR-18a-5p. High glucose-induced EndMT is also suppressed by miR-221/222 family, via the negative regulation of Wnt/β-catenin, and by miR-202-5p via inhibition of TGFβR2/TGFβ signaling pathway. Pro-fibrotic miRNAs are showed in dark, anti-fibrotic miRNAs in red.

**FIGURE 2:**
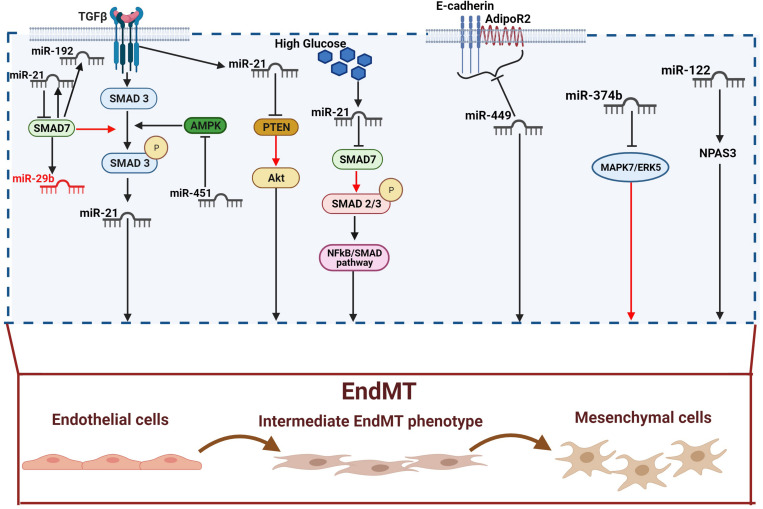
Pro-fibrotic miRNAs in diabetic complications. TGF-β increases miR-21 expression through Smad3 activation. miR-21 expression is also directly increased by TGF-β and high glucose. miR-21 can in turn activates EndMT through releasing PTEN of Smad7 inhibition (red arrow). Indeed, both PTEN and SMAD7 are negative regulators of EndMT via the Akt and TGF-β1/Smad3 signaling respectively. SMAD7 can also suppress fibrosis by down-regulating the pro-fibrotics miR-21 and miR-192, and up-regulating the anti-fibrotic miR-29b. miR451 triggers EndMT by blocking AMPK, an inhibitor of the TGF-β/SMAD pathway. miR-449a induces EndMT by inhibiting AdipoR2 and *E*-cadherin interaction in the lipid rafts. miR-374b plays its profibrotic activity by releasing MAPK7/ERK5-mediated EndMT inhibition. Finally, miR-122 activates EndMT via the neuronal PAS domain protein 3 (NPAS3). Pro-fibrotic miRNAs are shown in dark, anti-fibrotic miRNAs in red.

The ECM is a three-dimensional network of macromolecules (proteoglycans and fibrous proteins), present in all tissues and organs, that contributes to tissue morphogenesis, differentiation and homeostasis. Collagens, elastins, fibronectins, and laminins are the main proteins constituting the ECM (Frantz et al., 2010; Yue, 2014). The excessive deposition of ECM components is the hallmark of fibrosis, which represents a key pathophysiological step in many chronic inflammatory diseases, including diabetes (Herrera et al., 2018). Myofibroblasts are the main cellular mediators of fibrosis as they have the ability to invade the interstitial space and produce excessive amounts of ECM proteins (Zent and Guo, 2018). Although resident mesenchymal cells are the main source of myofibroblasts, the latter can also derive from other type of cells including pericytes, fibrocytes, epithelial and endothelial cells (ECs). The process involving ECs, known as EndMT, has been shown to actively contribute to the progression of renal fibrosis (Zeisberg et al., 2008; Curci et al., 2014; Sun et al., 2016). Besides, the mesenchymal shift contribution to kidney fibrosis can also be accelerate by the crosstalk between endothelium and epithelium, since EndMT can influence and induce EMT in tubular cells (Li et al., 2020b). In this context, *N*-acetyl-seryl-aspartyl-lysyl-proline (AcSDKP) plays a crucial role in inhibiting both EndMT and EndMT-mediated EMT. Its inhibitory action is exerted by targeting the fibroblast growth factor receptor 1 (FGFR1), an antifibrotic endothelial receptor (Li et al., 2020b), and by controlling the metabolic switch between glucose and fatty acid metabolism. Indeed, defects in normal kidney metabolism can accelerate EndMT and EndMT-mediated EMT contributing to kidney fibrosis (Srivastava et al., 2018, 2020b). An increasing body of evidence suggests that miRNAs are key regulators of EndMT as they appear differentially expressed under fibrotic stimuli such as high glucose, TGFβ, and hypoxia (Glover et al., 2019). This differential expression also reflects the specific role, profibrotic or antifibrotic, played by miRNAs (Hulshoff et al., 2019; Srivastava et al., 2019). The most potent inducer of kidney fibrosis is TGF-β (Wang J. et al., 2016; Wang Z. et al., 2017), which can trigger EndMT either by activation of specific signaling pathways, such as Akt and Smad (Wang J. et al., 2016; Wang Z. et al., 2017), or by increasing the expression of pro-fibrotic miRNAs (Srivastava et al., 2019). In this context, TGF-β mediates EndMT through the up-regulation of miR-21, a key modulator of fibrosis (Srivastava et al., 2013; Huang et al., 2015). Specifically, TGF-β elicits miR-21 increase through the activation of Smad3 which regulates miR-21 expression both at a transcriptional and a post-transcriptional level (Zhong et al., 2011). In addition, Smad3 modulates the expression of other miRNAs and activates the expression of various fibrotic genes (Loboda et al., 2016). Another mechanism used by miR-21 to stimulate renal fibrosis is the inhibition of Smad7 protein, a negative regulator of TGF-β1/Smad3 signaling. In this context, Smad7 has been shown to suppress renal fibrosis by down-regulating pro-fibrotic miRNAs such as miR-21 and miR-192 while up-regulating the anti-fibrotic miR-29b (Chung et al., 2013; Loboda et al., 2016). Additionally, miR-21 also regulates TGF-β-mediated EndMT through the PTEN/Akt pathway (Kumarswamy et al., 2012). Specifically, TGF-β increases the endothelial expression of miR-21, which in turn decreases the expression of PTEN, ultimately promoting EndMT by Akt activation (Meadows et al., 2009; Medici et al., 2011; Kumarswamy et al., 2012). Another molecule linked to TGF-β signaling in kidney fibrosis is the dipeptidyl peptidase-4 (DDP-4), a multi-functional protein expressed on the surface of most cell types, including ECs (Deacon, 2019). DPP-4 overexpression induces TGF-β-mediated EndMT in diabetic nephropathy (Shi et al., 2015; Kanasaki, 2016). Furthermore, recent studies have reported a relationship between DPP-4 and miR-29 in diabetic kidney fibrosis, where the overexpression of DPP-4 results associated with the suppression of miR-29s family anti-fibrotic activity (Kriegel et al., 2012; Harmanci et al., 2017). In line with these observations, the use of the DPP-4 inhibitor, linagliptin, ameliorates kidney fibrosis by restoring miR-29s and consequentially inhibiting EndMT in diabetic mice (Kanasaki et al., 2014). The anti-fibrotic peptide, AcSDKP which suppresses the TGF-β-induced EndMT in diabetic kidney (Nagai et al., 2014; Hrenak et al., 2015) can also, alone or in combination with angiotensin-converting enzyme inhibitor (ACEi), ameliorates renal fibrosis by suppressing DPP-4 and restoring the anti-fibrotic miR-29s and miR-let-7s expression in TGF-β-induced EndMT (Srivastava et al., 2020a). The crosstalk between miR-29s and miR-let-7s is crucial for maintaining endothelial cell homeostasis and AcSDKP potentiates this crosstalk regulation (Srivastava et al., 2019). Indeed, the presence of AcSDKP upregulates the antifibrotic miR-let-7 families, especially miR-let-7b, which suppress TGFβR1 and TGFβ signaling (Srivastava et al., 2016). Suppression of TGFβ signaling results in the up-regulation of the miR-29 family expression, which in turn induce FGFR1 phosphorylation, a critical step for miR-let-7 production (Srivastava et al., 2016, 2019). The associated expression of miR-29 and miR-let-7 is also regulated by an alternative mechanism involving interferon-gamma (IFNγ) (Srivastava et al., 2019). Precisely, miR-29 target the profibrotic IFNγ (Ma et al., 2011) blocking its inhibitory action toward FGFR1 which in turn induces the expression of miR-let-7 (Chen et al., 2012; Srivastava et al., 2019). Although not strictly related to DN, an additional anti-fibrotic mechanism, occurring by the suppression of DPP-4, involves miR-448-3p. EndMT inhibition and amelioration of vascular dysfunction has been indeed observed in both diabetic mice and cell models overexpressing miR-448-3p (Guan et al., 2020). A further regulatory mechanism of EndMT in diabetic nephropathy involves miR-497 and its two targets, ROCK1 and ROCK2, which belong to the rho-associated kinases (ROCKs) family and are activated in diabetes (Kolavennu et al., 2008; Liu et al., 2018; Matoba et al., 2020). A recent study showed that ROCKs inhibition, following treatment with melatonin (*N*-acetyl-5-methoxytryptamine), suppressed TGF-β2-induced EndMT. Specifically, the negative modulation of ROCK1 and ROCK2 is associated with the melatonin-induced up-regulation of miR-497, both in glomerular cells and diabetic rats (Liu et al., 2018). See figures and associated tables to overview of the signaling pathways involving both anti-fibrotic (Figure 1 and Table 1) and pro-fibrotic (Figure 2 and Table 2) miRNAs.

**TABLE 1:** Anti-fibrotic miRNAs in diabetic complications.

Anti-fibrotic miRNAs in diabetic complications					
miRNAs	DN	DR	Other	DCM	References
miR-142-3p				TGFβ-SMAD	Zhu et al., 2018
miR-200b miR-200b		TGFβ1-p300		TGFβ-p300	Cao et al., 2014; Feng et al., 2016
miR-202-5p		TGFβR2			Gu et al., 2020
miR-497b	ROCK1/2				Liu et al., 2018
miR-221/222 miR-221/222				Wnt-β/Catenin	Verjans et al., 2018; Wang et al., 2020
miR-29s miR-29s	TGFβ signaling	Notch2			Srivastava et al., 2016, 2019, 2020a; Zhang et al., 2019
miR-Let7	TGFβ signaling				Srivastava et al., 2016, 2019, 2020a
miR-448-3p			TGFβ signaling		Guan et al., 2020
miR-18a-5p				Notch2	Geng and Guan, 2017

*Summarizes the references describing the anti-fibrotic miRNAs in diabetic complication. DN, diabetic nephropathy; DR, diabetic retinopathy; DCM, diabetic cardiomyopathy.*

**TABLE 2:** Pro-fibrotic miRNAs in diabetic complications.

Pro-fibrotic miRNAs in diabetic complications					
miRNAs	DN	DR	AS	DCM	References
miR-21	TGFβ-SMAD				Srivastava et al., 2013
miR-21	PTEN/Akt				Kumarswamy et al., 2012
miR-21				NFkB/SMAD	Li Q. et al., 2020
miR-451				TGFβ-SMAD	Liang et al., 2019
miR-449			*E*-cadherin/AdipoR2		Jiang et al., 2019
miR-374b			MAPK7/ERK		Vanchin et al., 2019
miR-122			NPAS3		Wu et al., 2021

*Summarizes the references describing the pro-fibrotic miRNAs in diabetic complications. DN, diabetic nephropathy; DR, diabetic retinopathy; DCM, diabetic cardiomyopathy; AS, atherosclerosis.*

## Diabetic Cardiomyopathy

Diabetic cardiomyopathy (DCM), another common complication in diabetes, refers to myocardial dysfunction in the absence of conventional cardiovascular complications (coronary artery disease, valvular disease) and risk factors (hypertension, dyslipidemia) (Boudina and Abel, 2010; Jia et al., 2018). In the early stages, DCM is usually asymptomatic and characterized by left ventricular (LV) hypertrophy, LV diastolic dysfunction with diastolic filling abnormalities, myocardial fibrosis and cell signaling abnormalities. Disease progression leads to systolic dysfunction (left ventricular low ejection fraction) accompanied by heart failure, which is characterized by marked hypertrophy and fibrosis in the advanced stages (Boudina and Abel, 2010; Jia et al., 2018; Tan et al., 2020). Hyperglycemia, insulin resistance, lipid metabolism defects and oxidative stress up-regulate the production of advanced glycation end-products (AGEs) and Ang II, which in turn induce mitochondrial dysfunction in cardiomyocytes and ECs (Tan et al., 2002; Dikalov and Nazarewicz, 2013; Yan et al., 2014; Brunvand et al., 2017). Mitochondrial dysfunction, as well as the Ang II-induced NADPH oxidases stimulation, increases ROS production and oxidative stress (Dikalov and Nazarewicz, 2013; Siasos et al., 2018). Additionally, oxidative stress is also increased by lipid accumulation caused by an insulin resistance-induced cardiomyocytes metabolic shift. Indeed, the increased intake of fatty acid is not adequately metabolized by β-oxidation resulting in lipotoxicity (Boudina and Abel, 2010; Tan et al., 2020). Oxidative stress can in turn trigger endoplasmic reticulum (ER) stress, impairment of mitochondrial Ca^2+^ uptake, cardiomyocyte hypertrophy, ECs damage, microvascular dysfunction and the profibrotic responses by fibroblasts and inflammatory cells (Boudina and Abel, 2010; Tan et al., 2020). All these effects contribute to the accumulation of ECM, especially collagen type I and III, leading to myocardial fibrosis (Jia et al., 2018; Gollmer et al., 2019). The main signaling pathways underlying these pathophysiological events include TGFβ/SMAD, NFκB/SMAD, PKC, MAPK, Wnt/β-catenin, Notch2 and AcSDKP-FGFR1 signaling pathway (Nemir et al., 2014; Hu et al., 2018; Ma et al., 2018; Hortells et al., 2019; Li et al., 2020b; Yousefi et al., 2020). Most of these pathways lead to the development of cardiac fibrosis through the differentiation of fibroblasts into myofibroblasts as well as the endothelial-to-mesenchymal or epithelial-to-mesenchymal transition (Kovacic et al., 2012). Furthermore, increasing evidence suggests that miRNAs are the main players in the regulation of multiple pathways and cellular processes leading to cardiac fibrosis (Guo and Nair, 2017; Nandi and Mishra, 2018; Yousefi et al., 2020).

## miRNAs Regulation of DCM-Associated EndMT

The hyperglycemia-induced ECs damage and activation, resulting in vascular remodeling and EndMT, has been confirmed in myocardial fibrosis (Sharma et al., 2017). As suggested by experimental evidence, cardiac fibrogenesis involves the presence of a subset of EndMT-derived activated cardiac fibroblasts (Widyantoro et al., 2010; Sharma et al., 2017; Sánchez-Duffhues et al., 2018). Similarly, miRNAs are an important regulatory mechanism in cardiac fibrosis and heart failure (Wang Z. et al., 2016; Wang and Cai, 2017). In this context, miR-21, which has been widely described in pulmonary and renal fibrosis (Liu et al., 2016), plays an important role also in the pathogenesis of cardiac fibrosis and DCM (Adam et al., 2012; Guo and Nair, 2017; Yuan et al., 2017; Dai et al., 2018). A recent *in vivo* study confirmed the involvement of miR-21 in EndMT activation and myocardial fibrosis, showing that the hyperglycemia-induced up-regulation of miR-21 in diabetic mice is associated with the down-regulation of endothelial markers and the up-regulation of fibroblast markers (Li Q. et al., 2020). Moreover, similarly to the mechanism described in diabetic nephropathy (Zhong et al., 2011), miR-21 regulates EndMT through the NF-κB-SMAD signaling pathway by targeting SMAD7. The consequent SMAD7 inhibition increases SMAD2 and SMAD3 phosphorylation, resulting in EndMT activation (Li Q. et al., 2020). An additional mechanism, requiring the TGF-β/SMAD pathway, involves miR-142-3p, which has been shown to attenuate the hyperglycemia-induced EndMT in human aortic endothelial cells (HAECs) (Zhu et al., 2018). Indeed, miR-142-3p overexpression inhibits EndMT by inactivating both TGF-β1 and the downstream target gene SMAD2. By contrast, TGF-β1 overexpression significantly abolishes the inhibitory effects of miR-142-3p (Zhu et al., 2018). A negative regulation of glucose-induced EndMT in the heart is also played by miR-200b (Feng et al., 2016). In a recent study, the expression of specific fibrotic markers, such as vascular endothelial growth factor (VEGF) (Yang et al., 2014), zinc finger E-box–binding homeobox (Zeb2) (Jahan et al., 2018), and TGF-β1 (Biernacka et al., 2011) was prevented in diabetic mice overexpressing miR-200b (Feng et al., 2016). Moreover, miR-200b overexpression also induces the down-regulation of p300, a transcription coactivator known to contribute to cardiac fibrosis and hypertrophy via TGF-β/SMAD (Bugyei-Twum et al., 2014; Feng et al., 2016). Although the inhibitory role of the whole miR-200 family is well established, both in EMT (Korpal and Kang, 2008; Korpal et al., 2008) and EndMT (Feng et al., 2016; Zhang et al., 2017), unexpectedly a recent study shown that miR-200c-3p exerted the opposite effect, being able to promote EndMT and aortic graft remodeling both *in vivo* and *in vitro* (Chen et al., 2021). Finally, a further TGF-β/SMAD pathway-mediated regulatory mechanism involves miR-451 whose effects on EndMT are AMPK-dependent. Indeed, miR451 knockdown in diabetic mouse hearts suppresses EndMT through the activation of AMPK, which in turn inhibits the TGF-β/SMAD pathway (Liang et al., 2019). As previously mentioned, in addition to TGF-β/SMAD, other pathways underlie the pathophysiological events leading to cardiac fibrosis. One of them is the Wnt signaling pathway, known to promote fibroblast activation and proliferation (Tao et al., 2016). On the other hand, the anti-fibrotic role of miRNA-221/222 family has been confirmed, as their down-regulation was associated with heart failure (Verjans et al., 2018). The interplay between Wnt and miR-222 in EndMT regulation has been recently suggested (Wang et al., 2020); specifically, miR-222 is able to suppress the hyperglycemia-induced EndMT and inhibit cardiac fibrosis by negatively regulating the Wnt/β-catenin pathway in diabetic mice (Wang et al., 2020). Lastly, a further protective effect versus EndMT is exerted through the notch pathway and involves miR-18a-5p (Geng and Guan, 2017). The role of the notch pathway in heart development and control of the balance between fibrotic and regenerative repair in the adult heart has been widely confirmed (Nemir et al., 2014). Moreover, Notch2 activation results essential for driving ECs differentiation (Noseda et al., 2004; Kovacic et al., 2019) in cardiovascular disease and for promoting EndMT independently or in association with TGF-β/SMAD3 signaling (Fu et al., 2009; Chang et al., 2011). Notch2 is a target of miR-18a-5p which recently confirmed its antifibrotic role via the suppression of Notch2 and consequent inhibition of hyperglycemia-induced EndMT in human aortic valvular endothelial cells (HAVECs) (Geng and Guan, 2017). See figures and associated tables to overview of the signaling pathways involving both anti-fibrotic (Figure 1 and Table 1) and pro-fibrotic (Figure 2 and Table 2) miRNAs.

## Diabetic Retinopathy

Diabetic retinopathy (DR) is a common and severe microvascular complication of the eye that represents the leading cause of blindness in diabetes (Sabanayagam et al., 2016). The prevalence increases with disease progression and consequently with the exposure to the major risk factors, hyperglycemia and hypertension (Ding and Wong, 2012; Lee et al., 2015). Generally, a tight blood glucose control is cornerstone to reduce the risk of DR progression (Cheung and Wong, 2008). The condition is initially characterized by an asymptomatic stage, non-proliferative diabetic retinopathy (NPDR), that involves increased vascular permeability and capillary occlusion. Retinal neovascularization, by contrast, predominates in a later stage, proliferative diabetic retinopathy (PDR) (Lechner et al., 2017; Kusuhara et al., 2018), as consequence of hypoxia. However, as new vessels are relatively fragile, they tend to bleed into the macular region causing vision difficulties and, in the worst-case scenario, diabetic macular edema (DME), the main cause of blindness in DR (Wang and Lo, 2018). DME is described as a swelling of the macula due to fluid accumulation following breakdown of the blood-retinal barrier (BRB). This event can occur both in the PDR and in the NPDR stage (Das et al., 2015; Romero-Aroca et al., 2016). The BRB is composed of two distinct barriers: the outer BRB, consisting of retinal pigment epithelium and the inner BRB, composed of endothelial cells regulating the transport across retinal capillaries. Besides, the BRB is established by tight cellular junctions, both in the inner and outer barrier, as well as by the scarcity of endocytic vesicles within cells, which further ensure the integrity of the BRB (Klaassen et al., 2013; Díaz-Coránguez et al., 2017). In addition, pericytes, specialized mural cells with a central role in angiogenesis, regulate and stabilize this tight structure through the Angiopoietin-1/Tie-2, platelet-derived growth factor (PDGF) and TGF-β signaling pathways (Caporarello et al., 2019; Trost et al., 2019). BRB breakdown is a complex process involving different mechanisms; it can occur either in the inner BRB, the outer BRB, or both sites. The loss of integrity of the endothelial cell-cell junctions, the loss of pericytes and the thickening of the basement membrane are the major alterations observed in the inner BRB (Hammes et al., 2011; Das et al., 2015). Several studies have shown that hyperglycemia represents the main risk factor contributing to the pathogenesis of diabetic retinopathy (Engerman and Kern, 1986; Das et al., 2015; Eshaq et al., 2017). Furthermore, using a BRB model formed by retinal pericytes, astrocytes and endothelial cells, it has been recently reported that high glucose exposure elicits BRB breakdown, enhances BRB permeability and reduces the levels of junction proteins such as ZO-1 and VE-cadherin (Fresta et al., 2020). Besides, elevated ROS as well as pro-inflammatory mediators (IL-1β, IL-6) and oxidative stress-related enzymes (iNOS, Nox2) have also been shown to be increased (Fresta et al., 2020). The major biochemical pathways involved in the BRB breakdown are the polyol pathway, the AGEs pathway, the PKC pathway and the hexosamine pathway. Oxidative stress and inflammation are responsible for the upregulation of growth factors and cytokines, such as VEGF, tumor necrosis factor (TNF), interleukins (ILs), and matrix metalloproteinases (MMPs), which contribute to the BRB breakdown and to the development of DME (Aiello et al., 1994; Brownlee, 2005; Gupta et al., 2013; Das et al., 2015). Studies have confirmed the role of the pro-angiogenic factor VEGF as main modulator of PDR and DME. VEGF is secreted by retinal pigmented epithelial cells, pericytes, and endothelial cells in response to hypoxia conditions caused by the obstruction and loss of retinal capillaries (Gupta et al., 2013; Romero-Aroca et al., 2016). VEGF, in addition to promoting neovascularization in PDR, participates in the breakdown of the BRB via increasing permeability of retinal vessels (Ray et al., 2004). Indeed, high levels of VEGF increase the expression of the inflammatory intercellular adhesion molecule-1 (ICAM-1) which in turn facilitates the adhesion of leukocytes to the diabetic retinal vasculature, promoting capillary occlusion (Aiello et al., 1994; Joussen et al., 2002; Romero-Aroca et al., 2016).

## miRNAs Regulation of DR-Associated EndMT

Hyperglycemia-induced increased production of ECM and thickening of the vascular basement membrane is the hallmark of diabetic retinopathy (Roy et al., 2015). As previously mentioned, hyperglycemia promotes fibrosis progression through the generation of ECs-derived myofibroblasts, EndMT. This process has been shown to play an important role also in the pathogenesis of DR (Cao et al., 2014). Similar to other diabetic complications, TGF-β is an important EndMT mediator, mainly through the activation of the SMAD signaling pathways (Van Geest et al., 2010; Cao et al., 2014; Pardali et al., 2017). Moreover, the transcriptional activator p300, already known for increasing the expression of ECM proteins (Kaur et al., 2006), and miR-200b have been described as key regulators of the TGF-β-mediated EndMT in diabetic mice (Cao et al., 2014). Although the specific mechanism played by miR-200b and p300 remains partially unknown, the anti-fibrotic activity of miR-200b, already described in other diabetic complications (McArthur et al., 2011; Feng et al., 2016), has also been confirmed in DR. Specifically, the EndMT observed in the retinas of wild-type diabetic mice was suppressed by the overexpression of miR-200b (Cao et al., 2014). As mentioned before, the outer BRB is composed of tight junctions of retina pigment epithelial cells (RPECs) which secrete various factors, nutrients and signaling molecules that influence the surrounding tissues (Campbell and Humphries, 2013; Liu and Liu, 2019). Chronic hyperglycemia alters RPECs functions contributing to the fluid accumulation in DME and the development of DR (Desjardins et al., 2016). Under stress conditions RPECs cells can release large amounts of exosomes, nanoscale vesicles that mediate many intercellular activities such as cell-to-cell communication, immune regulation, inflammatory response, extracellular matrix turnover and neovascularization (Klingeborn et al., 2017; Liu et al., 2020). A recent study confirmed the importance of the crosstalk between ECs and RPECs cells in the progression of fibrosis in patients with DR (Gu et al., 2020). Specifically, it was observed that hyperglycemia increased the ability of RPECs to release miR-202-5p-enriched exosomes. On the other hand, hyperglycemia induced EndMT through the TGFβ signaling pathway activation in ECs. However, when ECs were treated with RPECs-derived exosomes, the hyperglycemia-induced TGFβ signaling pathway activation was significantly counteracted as well as the increased proliferation and migration (Gu et al., 2020). In addition, miR-202-5p, by targeting specifically TGFβR2, was responsible for the TGFβ signaling pathway inactivation and EndMT suppression (Gu et al., 2020). This study, in addition to providing additional evidence that hyperglycemia-induced EndMT involves the activation of TGFβ signaling, also showed that the release of miR-202-5p-enriched exosomes from RPE cells leads to the suppression of EndMT. The RPE cells-derived exosomes are therefore important mediators of the ECs-RPE cells crosstalk in the development of DR (Gu et al., 2020). Additional miRNAs involved in EndMT regulation in DR include two members of the mi-RNA29 family, miR-29a and miR-29b, already described in fibrosis development associated with diabetic complications (He et al., 2013; Kanasaki et al., 2014; Zhang Y. et al., 2014; Srivastava et al., 2020a). The anti-fibrotic activity of miR-29a/b has been recently confirmed also in DR where their overexpression suppressed the hyperglycemia-induced EndMT in human retinal microvascular endothelial cells (HRMECs) (Zhang et al., 2019). The inhibitory effect of miR-29a/b was exerted through the down-regulation of the transmembrane protein Notch2, known to activate morphological and functional changes of ECs as well as promote EndMT (Tian et al., 2017; Zhang et al., 2019). See figures and associated tables to overview of the signaling pathways involving both anti-fibrotic (Figure 1 and Table 1) and pro-fibrotic (Figure 2 and Table 2) miRNAs.

## Atherosclerosis

Atherosclerosis (AS) is characterized by plaque formation, secondary to the deposition of fats, cholesterol, and calcium, which lead to ischemia and its clinical manifestations, such as myocardial infarction and stroke (Lnsis, 2000). Although AS is classically associated with alterations of lipid metabolism and hypercholesterolemia (Wang H.H. et al., 2017), its pathogenesis is more complex and involves various factors. Endothelial dysfunction and inflammation are key steps in the sequence of events leading to AS (Davignon and Ganz, 2004; Hansson, 2009). The presence of mechanical stress, such as blood flow turbulence, can activate the endothelium, which responds by recruiting monocytes, adhesion molecules and pro-inflammatory cytokines. Monocytes, facilitated by adhesion molecules and cytokines, infiltrate the intima and can differentiate in macrophages which actively participate in lipid uptake through phagocytosis (Ilhan and Kalkanli, 2015). Diabetes and AS share several pathological mechanisms (La Sala et al., 2019b); indeed, the metabolic alterations that drive the development of diabetes are also involved in the pathogenesis of atherosclerosis (Federici and Lauro, 2005; Poznyak et al., 2020). In addition, both type 1 and type 2 diabetes can either induce atherosclerosis and accelerate its progression (Poznyak et al., 2020). In this context, a crucial role is played by the prolonged exposure to hyperglycemia and insulin resistance which are responsible for the increased atherosclerosis-related inflammation of the arterial wall (Reddy et al., 2010; Katakami, 2017). In addition to triggering the onset and progression of diabetes, insulin resistance also promotes dyslipidemia, hypertension and other metabolic abnormalities, important components of the pro-atherogenic milieu (Semenkovich, 2006; Katakami, 2017). At the same time, an insufficient insulin signaling elicits an abnormal lipid metabolism and glucose transport and increase the production of glucose in the liver. Pancreatic β cells respond to hyperglycemia by increasing insulin secretion; however, the continued stimulation of β cells leads to their progressive functional failure and diabetes development (Cavaghan et al., 2000; Mangiafico et al., 2011). Prolonged exposure to hyperglycemia increases oxidative stress (Yu et al., 2011; Volpe et al., 2018), the primary activator of signaling pathways driving AS and diabetes progression (Vanessa Fiorentino et al., 2013; Yuan et al., 2019). Overproduction of ROS increases the formation of AGEs, modifications of proteins or lipids that become non-enzymatically glycated (Moreno-Viedma et al., 2016; Katakami, 2017). AGEs are involved in each step of atherosclerosis, being responsible for monocyte migration into the sub-endothelial space, release of cytokines by macrophages and stimulation of vasoconstriction (Katakami, 2017). Moreover, the binding of AGEs to the receptor RAGE activates TGF-β, ERK, JNK, p38, NF-kB, PKC and the polyol pathways as well as maintaining the chronic pro-inflammatory state of the arterial wall (Katakami, 2017; Yamagishi and Matsui, 2018).

## miRNAs Regulation of AS-Associated EndMT

As previously mentioned, endothelial dysfunction driven by oxidative stress plays a critical role in the development of AS. Persistent activation of ECs induces EndMT, which contributes to both the initiation and the progression of atherosclerosis (Chen et al., 2015; Evrard et al., 2017). Moreover, the extent of EndMT in the human plaque appears to be strongly correlated with the severity of the disease (Souilhol et al., 2018). A recent study showed the up-regulation of 17 miRNAs in atherosclerotic plaques; among them, miR-449a, already known for its role in lipid and cholesterol anabolism as well as inflammation (Zhang H. et al., 2014), was significantly higher compared with normal arteries (Jiang et al., 2019). The authors reported that miR-449a induces EndMT and promotes the development of AS by targeting the interaction between adiponectin receptor 2 (AdipoR2) and *E*-cadherin in lipid rafts (Jiang et al., 2019). In this context, miR-449a has displayed a multilevel and complex regulatory mechanism by promoting proliferation and enhancing the migrating ability of ECs as well as their expression of atherosclerotic markers (Jiang et al., 2019). The ability to induce EndMT was confirmed by the reduced *E*-cadherin expression concurrently with the increased expression of α-SMA and SMAD3 (Jiang et al., 2019). miR-449a pro-atherosclerotic properties are exerted by inhibition AdipoR2 and *E*-cadherin migration into the lipid raft fractions of ECs and consequent suppression *E*-cadherin-AdipoR2 of interaction. Additionally, the authors reported that blocking miR-449a protects diabetic mice from developing AS (Jiang et al., 2019). Similarly to miR-449a, miR-374b was reported to be up-regulated both in atheroprone regions from mice and pigs and in TGF-β1-treated ECs (Vanchin et al., 2019). Additionally, the overexpression of miR-374b was associated with a reduction in endothelial markers (VE-Cadherin and eNOS), and a concomitant increase of mesenchymal markers (TAGLN and Calponin). Besides, miR-374b was able to induce EndMT through the silencing of the Mitogen-Activated Protein Kinase 7 (MAPK7) also known as ERK5 (Vanchin et al., 2019). MAPK7 is an antagonist of EndMT and its signaling activity is generally lost in vessel areas that are undergoing pathological remodeling (Nithianandarajah-Jones et al., 2014; Krenning et al., 2016). Similarly, MAPK7 signaling activity was lost in the sites of vascular remodeling, providing an additional confirmation of the inhibitory action of miR-374b. By contrast, the recovery of MAPK7 signaling abrogated the pathological effect of miR-374b (Vanchin et al., 2019). miR-122, another miRNA recently reported as EndMT mediator in AS, has been shown to be up-regulated both in the aortic intima of diabetic mice and in the cellular EndMT model (Wu et al., 2021). The regulatory action of miR-122 is mediated by the neuronal PAS domain protein 3 (NPAS3). Indeed, inhibition of miR-122 prevented atherosclerosis and regulated NPAS3-mediated EndMT (Wu et al., 2021). miR-122 might therefore represent a druggable target in preventing EndMT-associated atherosclerosis. See figures and associated tables to overview of the signaling pathways involving both anti-fibrotic (Figure 1 and Table 1) and pro-fibrotic (Figure 2 and Table 2) miRNAs.

## Long Non-Coding RNAs Regulation in Diabetes-Associated EndMT

Besides miRNAs, small RNAs also include long non-coding RNAs (lncRNAs) and circular RNAs (circRNAs) which are emerging as key regulators implicated in a significant number of biological processes (Qu et al., 2017; Statello et al., 2020). Unlike linear RNAs, circRNAs form a covalently closed continuous loop, without 5′ or 3′ ends (Qu et al., 2015). lncRNAs are instead linear RNAs, with a nucleotide length > 200, that can affect gene transcription both at the epigenetic, transcriptional and post-transcriptional level (Dykes and Emanueli, 2017; Wang C. et al., 2017). Thus, lncRNAs can differently interact with mRNAs, proteins, and DNA elements; moreover, the binding of transcriptional factors to the lncRNA promoter’s target sites can regulate their expression (Taft et al., 2010). lncRNAs are also precursors of many types of miRNAs, although more frequently they overlap both physically and functionally with the latter. Moreover, lncRNAs compete with miRNAs for the binding to the same target genes and can trigger miRNAs degradation (Taft et al., 2010; Chen et al., 2018). Hence, lncRNAs are involved in a variety of human diseases where they appear differentially expressed or genetically perturbed (Harries, 2012; Shi et al., 2013). In this context, most of the knowledge pertaining to lncRNAs is derived from cancer however there is increasing evidence of their involvement in other conditions, such as Alzheimer’s disease, diabetes, cardiac complications (DiStefano, 2018; Greco et al., 2018; Leung and Natarajan, 2018) and fibrosis (He Z. et al., 2020; Li et al., 2020a; Lin J. et al., 2020). One important function of lncRNAs is their role as a molecular sponge to certain miRNAs, hindering their expression (Biswas et al., 2018). This mechanism has been confirmed in diabetic kidney fibrosis, where the down-regulation of the anti-fibrotic miR-29 was associated with lncRNA H19 up-regulation, whereas its knockdown restored miR-29 activity and significantly inhibited TGF-β2-induced EndMT in diabetic mice (Shi et al., 2020). However, the role of H19 in diabetes-associated EndMT remains unclear; indeed, H19 overexpression prevented glucose-induced EndMT by reducing the TGF-β1 levels in DR (Thomas et al., 2019). Further studies are required to clarify the role of H19 in regulating EndMT in diabetic conditions. Another lncRNA involved in DR is the maternally expressed gene 3 (MEG3) which showed an inhibitory effect on hyperglycemia-induced EndMT. MEG3 resulted indeed able to suppress EndMT both *in vivo* and *in vitro* by inhibiting the PI3K/AKT/mTOR signaling pathway (He Y. et al., 2020). On the other hand, MEG3 methylation mediated by DNA methyltransferase 1 (DNMT1) attenuated MEG3 expression and consequently accelerated EndMT (He Y. et al., 2020). This finding clarifies the role of MEG3 in EndMT and provide additional confirmation that increased levels of DNA methylation represent a potential risk factor for the development of DR (Maghbooli et al., 2015). As previously reported, oxidized low density lipoproteins (ox-LDL), being able to trigger plaque formation and EndMT, are key players in AS development (Su et al., 2018). A recent study reported that miR-30c-5p and LINC00657, also known as non-coding RNA activated by DNA damage (NORAD), are both involved in ox-LDL-induced EndMT but with opposite effects (Wu et al., 2020). miR-30c-5p inhibited ox-LDL-induced EndMT via activation of the Wnt7b/β-catenin pathway whereas LINC00657, acting as sponge of miR-30c-5p, suppressed the EndMT inhibition (Wu et al., 2020). Indeed, the expression level of LINC00657 resulted elevated both in sera from AS patients and in ox-LDL-stimulated ECs (Wu et al., 2020).

## Potential ROS-EndMT-Small RNAs Interplay in Diabetes-Associated Fibrotic Conditions

Oxidative stress is a key player in the diabetic complications’ pathophysiology described in this review. Hyperglycemia is not only the main factor responsible for the increase in ROS but also favors the increase of inflammatory mediators, which ultimately leads to vascular dysfunction (Luc et al., 2019). Both genetic and epigenetic factors can regulate the development and exacerbation of oxidative stress; in this context, different studies have highlighted the key role played by miRNAs (Grieco et al., 2019). Indeed, hyperglycemia can alter miRNAs expression, which in turn contributes to the development of endothelium dysfunction and diabetic vascular disease (Luc et al., 2019). Besides, in diabetic complications the molecular mechanisms and signaling pathways triggered by oxidative stress appear similar to those involved in miRNAs regulation (Grieco et al., 2019; Qadir et al., 2019). Finally, hyperglycemia-induced oxidative stress can affect the expression of specific miRNAs, which in turn can exacerbate oxidative stress, in addition to regulating the fibrotic process through the mechanisms summarized in this review (Grieco et al., 2019; Qadir et al., 2019). On the other hand, oxidative stress is emerging as a key trigger of EndMT (Montorfano et al., 2014; Thuan et al., 2018). Therefore, although a direct oxidative stress-small RNAs-EndMT link has not been demonstrated in diabetes yet, a substantial body of evidence supports this interplay. For example, an indirect proof of a ROS-miR-21-EndMT link has been reported with kallistatin, an endogenous protein with beneficial effects on EndMT-associated fibrosis (Guo et al., 2015). Kallistatin treatment blocked TGF-β-induced EndMT, NADPH oxidase-dependent ROS formation and the expression of the pro-fibrotic miR-21, confirming the role of both miR-21 and ROS as major mediators of EndMT (Guo et al., 2015). Many studies indicated a direct link between mi-R21 and oxidative stress in diabetic subjects, where ROS generation has been suggested as a downstream effect of miR-21 overexpression (La Sala et al., 2019a). The pro-oxidant effect of miR-21 is exerted through the suppression of genes which usually limit oxidative damage such as KRIT1 (Krev/Rap1 Interaction Trapped-1), Nuclear Factor erythroid Related Factor 2 (NRF2), and MnSOD2 (Manganese-dependent Superoxide Dismutase2). By contrast, inhibition of miR-21 decreases ROS levels (La Sala et al., 2018; Grieco et al., 2019). A relationship between up-regulation of miR-21 and increased ROS levels has also been shown during the development of diabetic cardiac dysfunctions (Yildirim et al., 2013). The miR-200 family, the anti-fibrotic activity of which has been described both in diabetic nephropathy and retinopathy, has also been shown to be associated with a decrease in oxidative stress in diabetes; specifically, the antioxidant effect of miR-200 is exerted by silencing the O-GlcNAc transferase, also known as OGT, whose enzymatic activity is associated with diabetic complications and endothelial inflammation (Qadir et al., 2019). Another proof of the oxidative stress-small RNAs-EndMT interconnection comes from a study investigating the activity of miR-451 (Ruknarong et al., 2021). The latter, previously described for its ability to induce EndMT in diabetic mouse heart (Liang et al., 2019), has been recently reported to be up-regulated in diabetic subjects with high oxidative stress. The association between miR-451 and oxidative stress has been further confirmed with the use of the antioxidant Vitamin C; indeed, Vitamin C administration in diabetic subjects decreased both the expression of miR-451 and ROS levels (Ruknarong et al., 2021). Finally, an interplay being the basis of mitochondrial functions in kidney ECs involves the miR-let-7 family, (FGF)/FGFR1 signaling pathway and SIRT3 (Srivastava et al., 2020c). The integrity of the FGFR1-miR-let-7 axis, on which depends the modulation of SIRT3, is crucial for maintaining the mitochondrial functionality (Srivastava et al., 2020c). SIRT3, for its part, controls mitochondrial redox homeostasis by modulation of ROS levels (Jing et al., 2011; Bause and Haigis, 2013) mainly via activation of the antioxidant enzyme superoxide-dismutase 2 (Qiu et al., 2010). On the contrary, the loss of the FGFR1-miR-let-7axis impairs SIRT3 and miR-29 levels with consequent disruption of mitochondrial integrity and activation of pro-mesenchymal signaling (Wnt signaling, BMP, Notch, TGF-β signaling) promoting EndMT (Srivastava et al., 2020c).

## Conclusion and Future Directions

This review has highlighted the key role of EndMT in the fibrotic process occurring in the development of the major diabetic complications. Environmental factors (high glucose, hypoxia, oxidative stress, pro-inflammatory cytokines) are important determinants of EndMT induction through the activation of specific signaling pathways, such as TGF-β, Notch, Wnt, and the modulation of the expression of microRNAs. The evidence reviewed in this article indicates that some microRNAs, e.g., miR-29, miR-200, and miR-Let7, have anti-fibrotic effects and inhibit EndMT whereas others, e.g., miR-21 and miR-122, possess pro-fibrotic properties and promote EndMT. The anti-fibrotic activity of some microRNAs appears univocal not only within diabetic complications but also in other pathological conditions. For instance, miR-29a/b and miR-200b have been shown to inhibit fibrosis in pulmonary fibrosis (Yang et al., 2012; Cushing et al., 2015), systemic sclerosis (Harmanci et al., 2017) as well as in DCM, DN, and DR (Cao et al., 2014; Kanasaki et al., 2014; Feng et al., 2016; Zhang et al., 2019). Similarly, miR-21 is generally up-regulated in different fibrotic diseases (Huang et al., 2015; Liu et al., 2016) as well as in diabetic complications such as DN, DR, and DCM (Srivastava et al., 2013; Chen et al., 2017; Li Q. et al., 2020). Moreover, since the expression levels of miR-21 in the plasma of diabetic patients were correlated with disease progression, miR-21 might be used as a marker of diabetes severity (Jiang et al., 2017). On the other hand, the function of other microRNAs is only partially established in *in vitro* models or in specific pathological conditions. Further, for some miRNAs the evidence is still controversial, such as the case of the lncRNA H19 which showed pro-fibrotic activity in DN (Shi et al., 2020) and an opposite effect in DR (Thomas et al., 2019). Additionally, since the markers for EndMT used in individual studies are often different, a complete understanding of the regulatory mechanisms played by miRNAs, or an exact comparison between them, is currently challenging. In this regard, future directions in the study of diabetic complications should involve (a) a thorough characterization of the mechanisms involved in the ROS-EndMT-small RNAs interplay and its relationship with the onset and severity of specific complications, (b) the conduct of epidemiological studies investigating the association between specific miRNAs and lncRNAs and metabolic control, surrogate markers of organ damage, and morbidity and mortality in patients with diabetes, and (c) the effects of specific pharmacological and non-pharmacological interventions targeting EndMT on the risk and progression of diabetic complications. Such studies might contribute to the identification of new diagnostic and therapeutic strategies to prevent or limit the structural and functional damage that leads to organ and system failure in diabetes.

## Author Contributions

RG, YMAA, and GP: conceptualization. GKN, AAM, and GP: resources. RG and YMAA: writing the original manuscript draft. RG, YMAA, HA, SA, LP, GKN, AAM, and GP: review and editing the different manuscript versions. AAM and GP: final editing and supervision. GP: submission. All authors: read and agreed to the published version of the manuscript.

## Conflict of Interest

The authors declare that the research was conducted in the absence of any commercial or financial relationships that could be construed as a potential conflict of interest.
